# Supplementary material for: Targeting the Tumor Microenvironment through mTOR Inhibition and Chemotherapy as Induction Therapy for Locally Advanced Head and Neck Squamous Cell Carcinoma: The CAPRA Study
Source: Cancers (Basel). 2022 Sep 17;14(18):4509. doi: 10.3390/cancers14184509 (PMC9496893; doi:10.3390/cancers14184509)
Supplement: Supplementary file 1 [file cancers-14-04509-s001.zip › cancers-1880622-supplementary.pdf]

Table S1. Patient demographics and baseline characteristics of patients treated at the dose of 30 mg/week of everolimus.

Characteristics	
**Number of patients enrolled**	N=3
**Age, median (year); [range]**	61 [55-62]
**Gender,** n (%)	
Male	3 (100)
**WHO performance status**, n (%)	
0	2 (66.7)
1	1 (33.3)
**Disease stage**, n (%)	
IVa	2 (66.7)
IVb	1 (33.3)
**T stage**, n (%)	
2	2 (66.7)
4a	1 (33.3)
**N stage**, n (%)	
2b	1 (33.3)
2c	1 (33.3)
3	1 (33.3)
**Primary site**, n (%)	
Oropharynx	2 (66.7)
Oral cavity	1 (33.3)
